# Sweat gland carcinoma with neuroendocrine differentiation (SCAND) arising in the axilla: A case report highlighting diagnostic challenges and surgical management

**DOI:** 10.1016/j.jpra.2026.01.013

**Published:** 2026-01-21

**Authors:** Maiko Inada, Takashi Nouchi, Yoshifumi Iwahashi, Miwako Miyasaka, Yutaka Inaba, Shinichi Asamura

**Affiliations:** a Department of Plastic and Reconstructive Surgery, Graduate School of Medicine, Wakayama Medical University, Wakayama, Japan; b Department of Human Pathology and Diagnostic Pathology, Graduate School of Medicine, Wakayama Medical University, Wakayama, Japan; c Department of Cardiovascular, Respiratory and Breast Surgery, Graduate School of Medicine, Wakayama Medical University, Wakayama, Japan; d Department of Dermatology, Graduate School of Medicine, Wakayama Medical University, Wakayama, Japan

**Keywords:** Sweat gland carcinoma with neuroendocrine differentiation (SCAND), Sweat gland carcinoma, Low-grade neuroendocrine carcinoma of the skin, (LGNECS)

## Abstract

**Background:**

Sweat gland carcinoma with neuroendocrine differentiation (SCAND) is a rare, newly recognized cutaneous adnexal tumor arising from sweat glands and characterized by neuroendocrine features. Given its rarity and resemblance to benign lesions, clinical diagnosis can be challenging.

**Case presentation:**

A 73-year-old man presented with a 40-year history of intermittent discharge from a right axillary mass, which had initially been diagnosed as an epidermal cyst. Following lesion excision along the tumor margin at a local clinic, histopathological analysis revealed apocrine carcinoma, and the surgical margin could not be definitively confirmed to be negative. He was then referred to our department, where positron emission tomography-computed tomography demonstrated abnormal uptake in the right axillary lymph nodes, with a maximum standardized uptake value of 5.70. We performed wide local excision with a 1-cm margin, as well as a level Ⅱ axillary lymph node dissection. Histopathological evaluation showed tumor infiltration with nodular and trabecular pattern in the dermis and the subcutaneous tissue. Tumor cells contained eosinophilic cytoplasm and round-shaped nuclei with granular chromatin. 28 lymph nodes were resected, among which 19 showed metastatic involvement. Immunohistochemistry showed positivity for GCDFP-15, GATA3, ER and neuroendocrine differentiation markers. These findings were consistent with SCAND. The wound was closed primarily without the need for flap reconstruction. There has been no evidence of recurrence or metastasis at 12 months of postoperative follow-up.

**Conclusion:**

This case highlights the potential for long-standing benign-appearing skin lesions to harbor rare malignant tumors such as SCAND. Accurate pathological diagnosis and increased clinical awareness among plastic surgeons are essential, and long-term surveillance is recommended owing to the possibility of delayed metastasis.

## Introduction

Sweat gland carcinoma with neuroendocrine differentiation (SCAND) is a recently proposed clinicopathological entity, first described by Goto et al. in 2017.^1^ It is a rare, low-grade malignant skin tumor that arises from sweat glands and exhibits neuroendocrine differentiation. In contrast to Merkel cell carcinoma and endocrine mucin-producing sweat gland carcinoma (EMPSGC), SCAND typically originates within the dermis, demonstrates indolent growth, and primarily affects middle-aged to elderly men along the embryological milk line, including the axilla and chest wall.^2^, ^3^, ^4^ Owing to the rarity of this condition, standard diagnostic and therapeutic protocols have not been established yet.

## Case presentation

**Figure 1:**
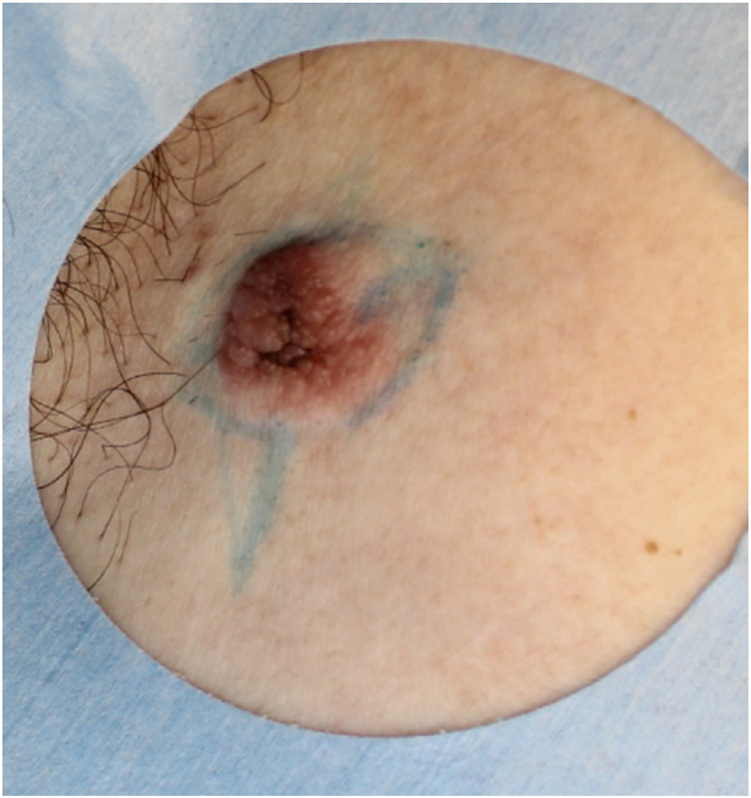
A 15 mm tumor was observed in the right axilla. It showed a reddish irregular-shaped tumor with mulberry-like surface.

The patient was a 73-year-old man with more than a 40-year history of intermittent purulent discharge from a right axillary skin mass, which had been clinically diagnosed as an epidermal cyst. The lesion was surgically excised at a local clinic (Figure 1). However, histological examination revealed apocrine adenocarcinoma, prompting referral to our department for further treatment.

**Figure 2:**
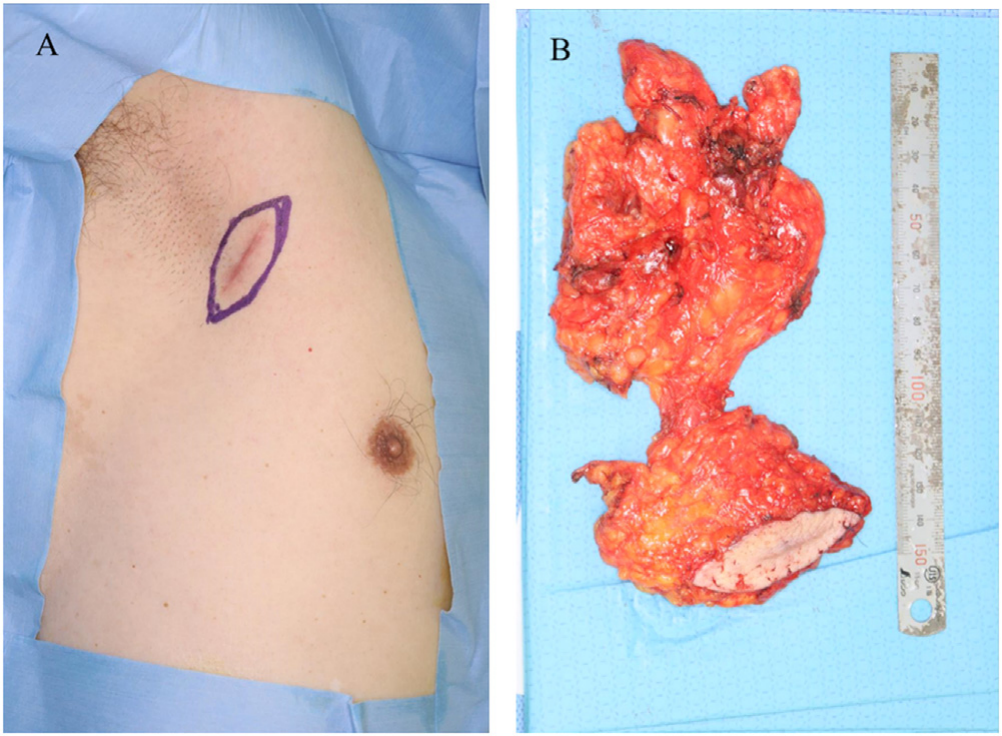
Wide local excision with a 1-cm margin was performed, along with axillary lymph node dissection.

At our institution, positron emission tomography-computed tomography showed abnormal fluorodeoxyglucose uptake in the right axillary lymph nodes (Supplementary material 1). Physical examination revealed a linear scar. Wide local excision with a 1-cm margin was performed, along with axillary lymph node dissection (Figs. 2). The surgical wound was closed primarily, without the need for skin flap reconstruction.

**Figure 3:**
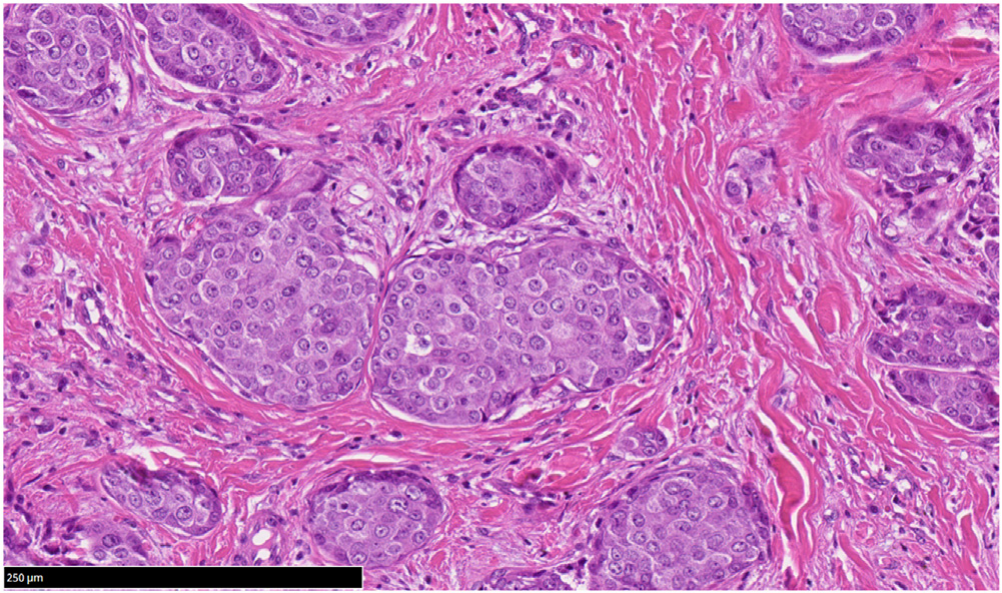
Tumor cells contained eosinophilic cytoplasm and moderately atypical nuclei with salt-and-pepper pattern chromatin. Necrosis was absent and mitotic figures were rare. No mucin production was identified. (PAS staining, × 200).

Histopathological analysis revealed nodular and trabecular pattern infiltration of tumor cells extending from the dermis to the subcutaneous tissue. Tumor cells contained eosinophilic cytoplasm and moderately atypical nuclei with salt-and-pepper pattern chromatin. Necrosis was absent and mitotic figures were rare. No mucin production was identified on PAS staining(Figure 3). Immunohistochemically, the tumor cells were positive for Ber-EP4, GCDFP-15, GATA3, ER, PgR, synaptophysin, chromogranin A, INSM1 (Supplementary material 2). Merkel cell polyomavirus antigen and c-kit were negative. We ruled out EMPSGC and Merkel cell carcinoma.^3^^,^^4^ Consequently, The final diagnosis was SCAND.^1^^,^^2^

No local recurrence or distant metastasis has been noted during 1 year of postoperative follow-up.

## Discussion

SCAND is a rare cutaneous adnexal carcinoma with neuroendocrine differentiation, first defined by Goto et al. in 2017.^1^ It differs from other neuroendocrine skin tumors in its relatively low malignant potential and exclusive dermal origin. In a 2022 case series of 13 patients, SCAND was reported to occur predominantly in elderly men, typically presenting as painless nodules on the chest or axilla—regions corresponding to the embryological milk line.^2^

Histologically, SCAND is characterized by medium-sized tumor cells arranged in solid nests or trabeculae, sometimes with focal ductal differentiation.^1^^,^^2^ Immunohistochemical positivity suggesting for both sweat-gland differentiation (such as GCDFP-15, GATA3, ER) and neuroendocrine differentiation (chromogranin A, synaptophysin, INSM1) is essential for diagnosis. The absence of mucin production (as seen in EMPSGC) and negative staining for CK20 (as seen in Merkel cell carcinoma) are useful for differential diagnosis.^3^^,^^4^

In Goto’s report, tumors smaller than 2 cm were associated with lymph node metastasis in fewer than 20% of cases. In contrast, all tumors over 2 cm showed nodal involvement, and tumors larger than 3 cm were associated with fatal outcomes.^2^ In our case, despite the small tumor size (<2 cm), lymph node metastasis was present. This may be attributed to the prolonged natural history—over 40 years—suggesting that not only tumor size but also duration of untreated disease may influence metastatic risk.

Although primary closure was sufficient in this case, awareness of SCAND is essential for plastic surgeons who may encounter such indolent but potentially malignant lesions misdiagnosed as benign cysts or scars. Proper oncologic excision and histopathological evaluation are mandatory for accurate diagnosis and treatment.^5^

## Conclusion

This case emphasizes the importance of considering rare malignant tumors such as SCAND in the differential diagnosis of long-standing skin lesions, particularly in the axilla. Plastic surgeons should be aware of the potential for misdiagnosis and ensure histological confirmation even in clinically benign-appearing lesions. Long-term follow-up is warranted, given the risk of delayed lymphatic metastasis.

## Funding

None.

## Appendix Supplementary materials

Image, application 1
