# Supplementary material for: Sweat gland carcinoma with neuroendocrine differentiation (SCAND) arising in the axilla: A case report highlighting diagnostic challenges and surgical management
Source: JPRAS Open. 2026 Jan 21;48:857–61. doi: 10.1016/j.jpra.2026.01.013 (PMC12924892; doi:10.1016/j.jpra.2026.01.013)
Supplement: Supplementary file 1 [file mmc1.docx]

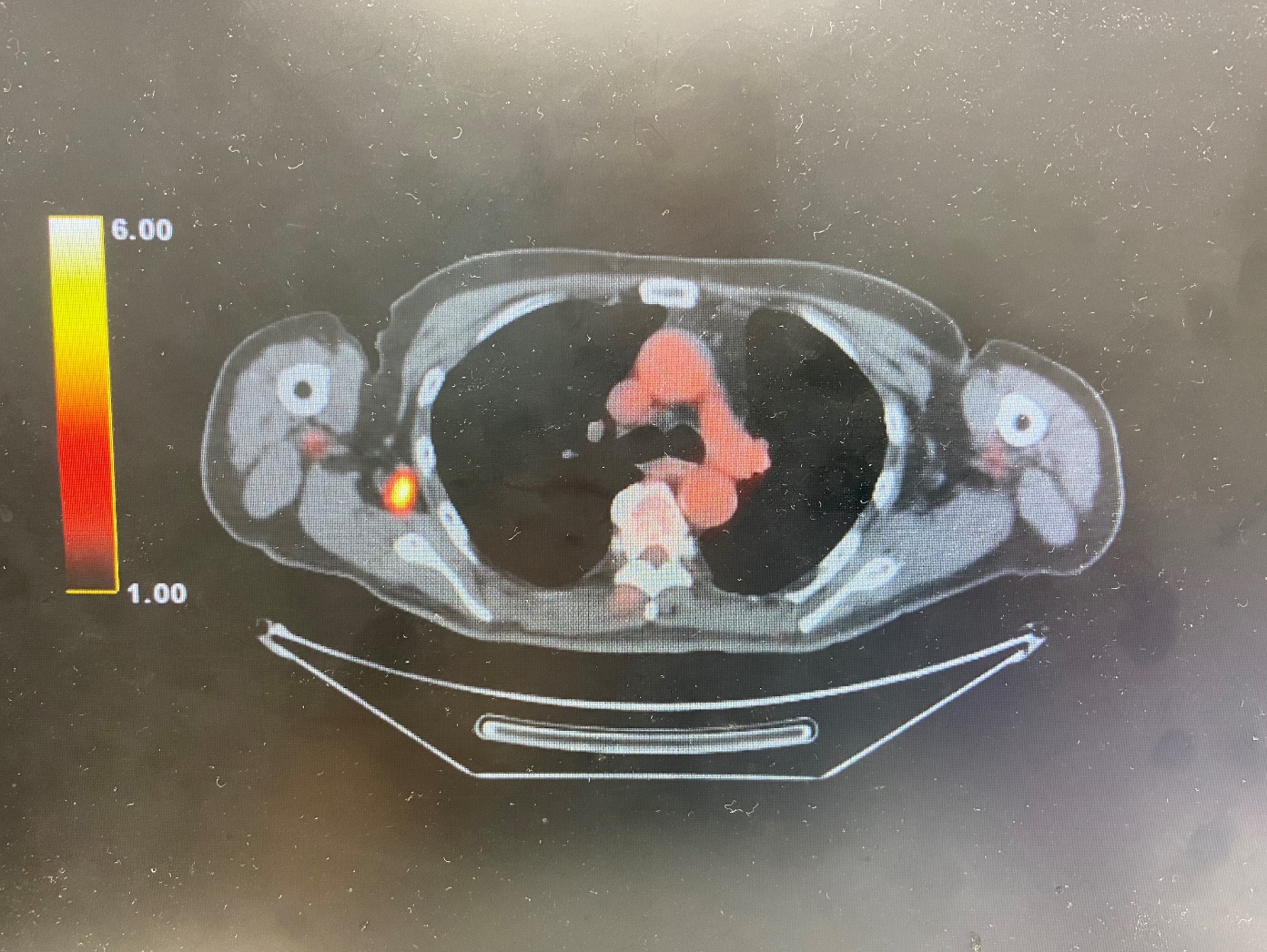


**Supplementary material 1.** Positron emission tomography-computed tomography showed abnormal fluorodeoxyglucose uptake in the right axillary lymph nodes


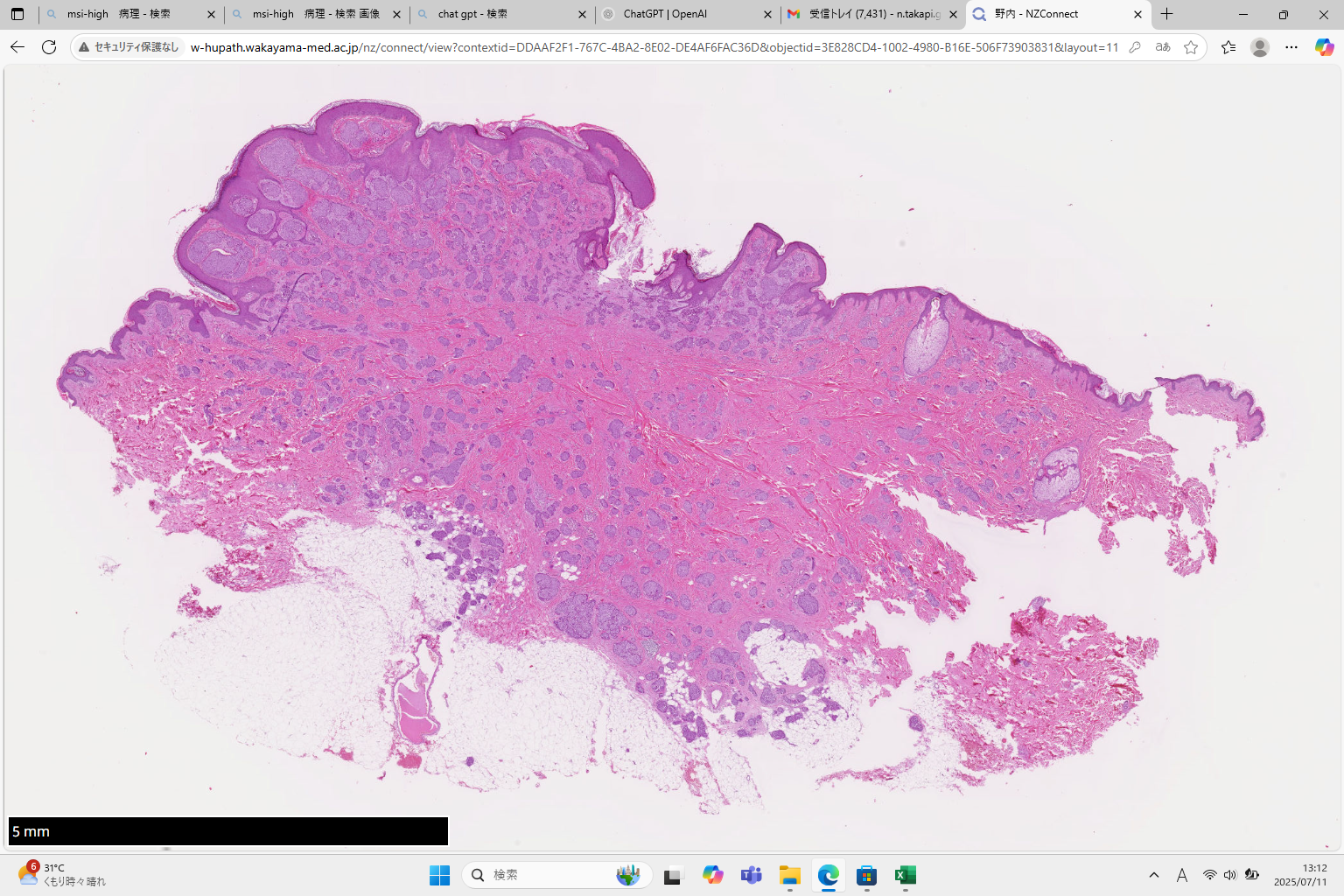


**Supplementary material 2:** Histopathological analysis revealed nodular and trabecular pattern infiltration of tumor cells extending from the dermis to the subcutaneous tissue. （PAS staining, ×10）


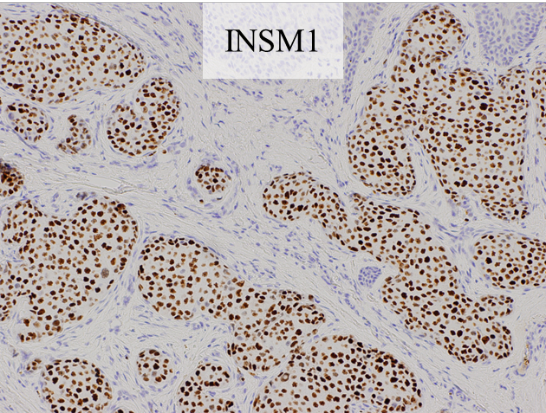

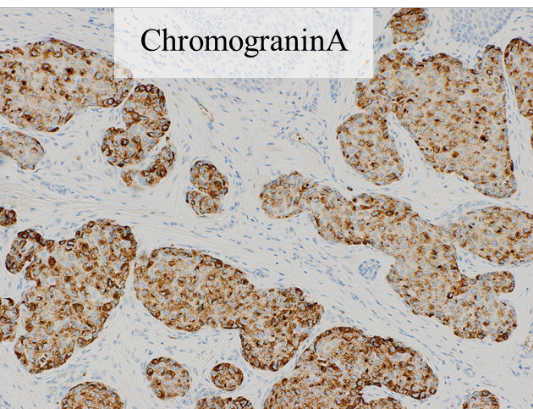

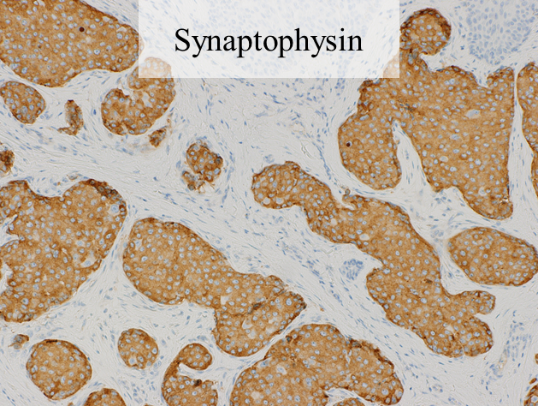

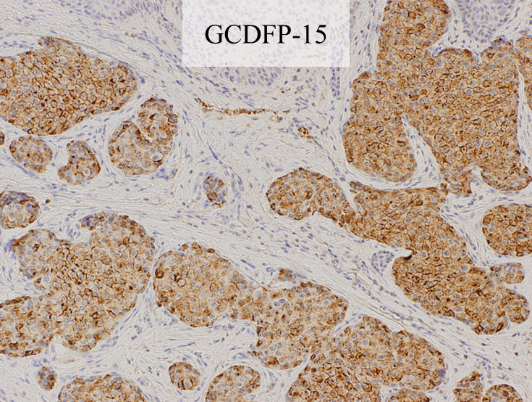

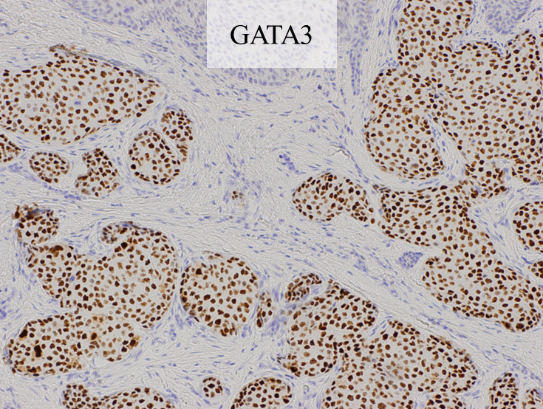


Immunohistochemically, the tumor cells were positive for GCDFP-15, GATA3, synaptophysin, chromogranin A, INSM1.


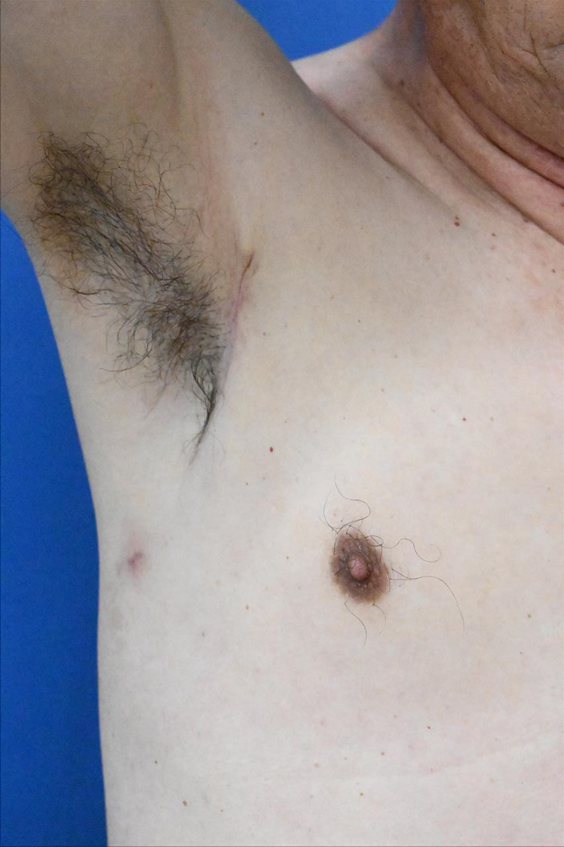


**Supplementary material 3:** Six months after surgery
